# Delayed expression of activity-dependent gating switch in synaptic AMPARs at a central synapse

**DOI:** 10.1186/s13041-019-0536-2

**Published:** 2020-01-15

**Authors:** Lee Stephen Lesperance, Yi-Mei Yang, Lu-Yang Wang

**Affiliations:** 1 Program in Neurosciences & Mental Health, SickKids Research Institute, 555 University Ave, Toronto, Ontario M5G 1X8 Canada; 2 Department of Biomedical Sciences, University of Minnesota, Duluth, MN 55812 USA

**Keywords:** Activity-dependent plasticity, Synaptic transmission, Developing plasticity, AMPAR subunit composition, Calyx of held-MNTB synapse

## Abstract

Developing central synapses exhibit robust plasticity and undergo experience-dependent remodeling. Evidently, synapses in sensory systems such as auditory brainstem circuits mature rapidly to achieve high-fidelity neurotransmission for sound localization. This depends on a developmental switch in AMPAR composition from slow-gating GluA1-dominant to fast-gating GluA4-dominant, but the mechanisms underlying this switch remain unknown. We hypothesize that patterned stimuli mimicking spontaneous/sound evoked activity in the early postnatal stage drives this gating switch. We examined activity-dependent changes in evoked and miniature excitatory postsynaptic currents (eEPSCs and mEPSCs) at the calyx of Held synapse by breaking through the postsynaptic membrane at different time points following 2 min of theta burst stimulation (TBS) to afferents in mouse brainstem slices. We found the decay time course of eEPSCs accelerated, but this change was not apparent until > 30 min after TBS. Histogram analyses of the decay time constants of mEPSCs for naive and tetanized synapses revealed two populations centered around τ_fast_ ≈ 0.4 and 0.8 ms, but the relative weight of the τ_0.4_ population over the τ_0.8_ population increased significantly only in tetanized synapses. Such changes are blocked by NMDAR or mGluR1/5 antagonists or inhibitors of CaMKII, PKC and protein synthesis, and more importantly precluded in GluA4^−/−^ synapses, suggesting GluA4 is the substrate underlying the acceleration. Our results demonstrate a novel form of plasticity working through NMDAR and mGluR activation to trigger a gating switch of AMPARs with a temporally delayed onset of expression, ultimately enhancing the development of high-fidelity synaptic transmission.

## Introduction

Synaptic development is traditionally believed to involve an early phase of genetically directed wiring, followed by the refinement of these connections through sensory activity. A growing number of observations suggest extensive cross-talk between genetic programs and neural activity is crucial to circuit organization before the onset of sensory inputs. Transiently observed patterns of spontaneous activity occur in various developing circuits, including the retina, cochlea, hippocampus and cerebellum, where it guides the wiring and tuning of neuronal connections early in development [1]. Prior to the onset of hearing, brief mini-bursts of spontaneous high-frequency spike discharges (up to several hundred hertz) separated by long latencies (in seconds) have been observed in vivo from the auditory brainstem [2–4] suggesting an important role for patterned activity in promoting synapse development in these circuits potentially by remodeling of composition of postsynaptic glutamate receptors.

Previous studies at the calyx of Held - principle neuron synapse in the medial nucleus of trapezoid body (MNTB), a glutamatergic synapse involved in the detection of interaural timing and intensity differences in the sound localization circuit, demonstrated a reorganization of postsynaptic glutamate receptors within the first 2 weeks of postnatal development. These processes involve a reduction in NMDA receptors (NMDARs) [5–7] paralleled by a switch from slow-gating GluA1-dominant AMPA receptors (AMPAR) to fast-gating GluA4-dominant AMPARs [8–11]. These alterations in glutamate receptors occur following hearing onset around postnatal day 11/12 (P11/12) and contribute to the characteristic ultra-fast EPSCs at mature calyx of Held-MNTB synapses [6, 8, 11, 12]. Although it is known that the AMPAR gating switch facilitates faithful high-frequency neurotransmission [8, 11], the critical link between activity and developmental gating switch in synaptic AMPARs remains undetermined.

The activation of NMDARs and Group 1 mGluRs is associated with the induction of various forms of synaptic plasticity [13, 14]. In the developing MNTB, Group 1 mGluRs and NMDARs are predominantly localized to the peri−/extrasynaptic regions [15] making them ideal sensors of glutamate spill-over induced by repetitive, high-frequency neural activity. By mimicking spontaneous discharge in vitro, our previous work at the calyx of Held-MNTB synapse [12] demonstrated that a 2-min theta burst stimulation (TBS) paired with postsynaptic depolarization coincidentally activated Group 1 mGluRs and NMDARs, and acutely induced peri−/extrasynaptic NMDAR endocytosis. As a consequence, neurotransmission fidelity was significantly enhanced, modeling developmental down-regulation of NMDARs observed in MNTB neurons following the opening of the ear canals. However, acceleration in AMPAR kinetics was never observed following TBS as would have been expected if the switch from GluA1- to GluA4-dominant receptors occurred in parallel.

In this study, we used the postsynaptic cell-attached configuration to minimize perturbations to intracellular signaling and revealed that application of the same paradigm as in our previous study to presynaptic axons results in an accelerated decay time course of eEPSCs and mEPSCs, only if membrane integrity is maintained for more than 30 min following TBS. Analysis of individual mEPSC decay constants reveal two mEPSC populations, one population with an average fast decay constant of 0.4 ms (τ_0.4_) and the other population with a fast time constant centered around 0.8 ms (τ_0.8_), in line with homomeric GluA4 and GluA1 values respectively. TBS increases the relative weight of the τ_0.4_ population at the expense of the τ_0.8_ population, suggesting activity drives the recruitment of GluA4 to replace GluA1 at the synapse and consequentially accelerate the AMPAR-EPSC time course.

## Methods

### Brainstem slice preparation

Mice were housed in a facility certified by the Canadian Council of Animal Care and used for this study in accordance with a protocol approved by the *Hospital for Sick Children Animal Care Committee.* The generation of AMPAR subtype 4 mice (GluA4^−/−^) and confirmation of deletion had been previously described [16]. Brainstem slices were prepared from P7–P10 CD1/C57 mice of either sex. Brains were dissected out of the animal then immersed in ice-cold artificial CSF (aCSF) containing (in mM) 125 NaCl, 2.5 KCl, 2 Na-pyruvate, 10 glucose, 1.25 NaH_2_PO_4_, 3 myo-inositol, 0.5 ascorbic acid, 26 NaHCO_3_, 3 MgCl_2_, and 0.1 CaCl_2_ at a pH of 7.3 when bubbled with 95% O_2_ and 5% CO_2_. The brainstem was glued, rostral side down, in the sectioning chamber of a vibratome (Leica VT1200S, Wetzler, Germany) and immersed in ice-cold, oxygenated aCSF. Three sequential transverse sections of the auditory brainstem were cut and placed in an oxygenated incubation tray at 35 °C for 1 h, and kept at room temperature thereafter for experiments.

### Electrophysiology

**Fig. 1:**
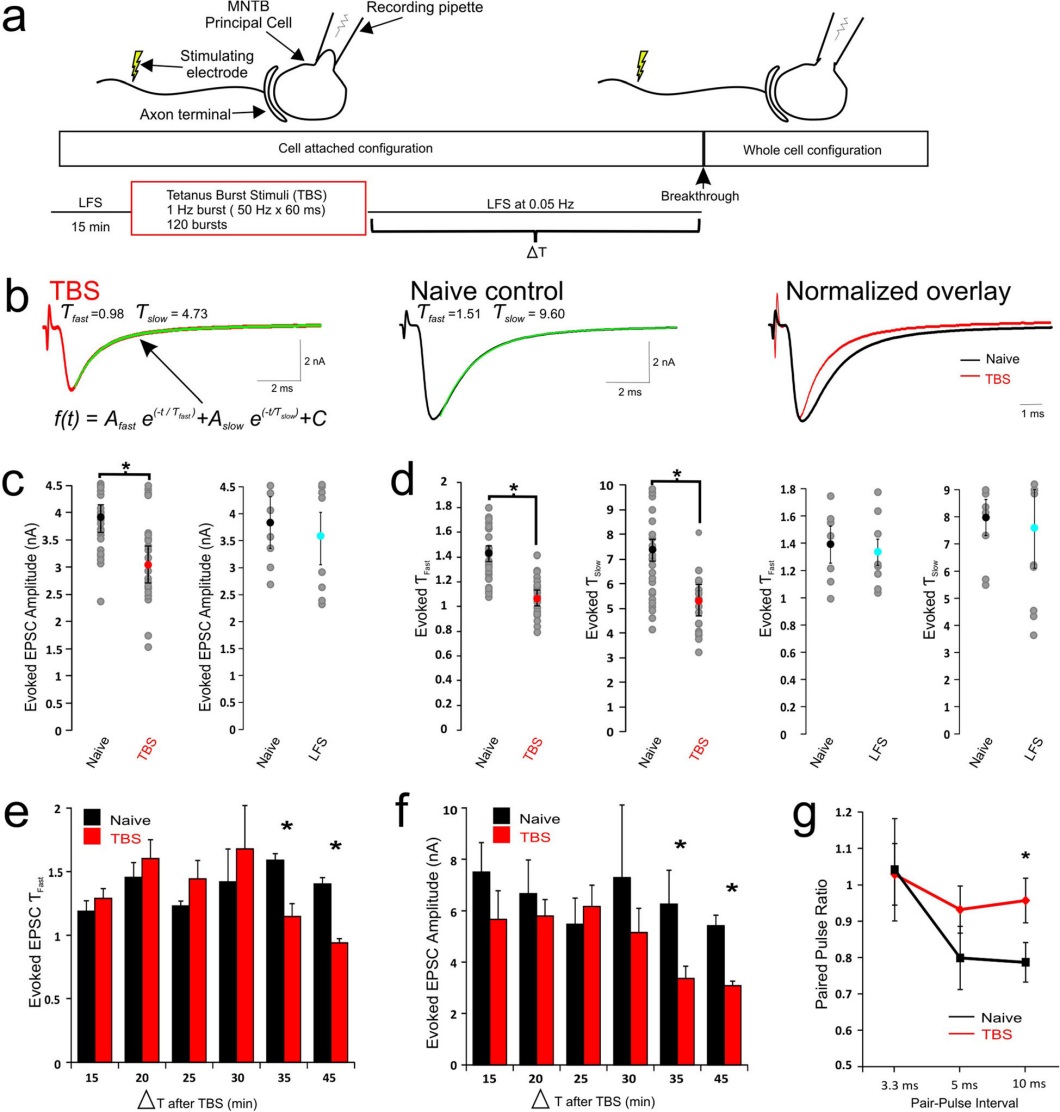
Delayed expression of activity-dependent acceleration in the kinetics of eEPSCs. **a** Schematic diagram shows details of experimental paradigm for induction and expression phases in cell-attached configuration before establishing whole-cell recording mode to measure eEPSCs (or mEPSCs) at different time points (Δt) after theta burst stimulation (TBS). Low frequency stimulation (LFS, 0.05 Hz) was given throughout experiments except for the period of TBS application. Parameters for TBS are given in the box. Control experiments were performed in contralateral MNTB nuclei of the same slices (naïve) where cells experienced no TBS prior to membrane rupture. **b** Examples of whole-cell recordings of averaged eEPSCs from naïve (*middle panel*) and TBS (*left panel*) synapses at Δt = 45 min, for which the decay phase is fit with a double exponential curve function with respective fast and slow time constants given (τ_fast_ and τ_slow_). Scaled eEPSCs from naive and TBS synapses are superimposed to illustrate the accelerated time course of synaptic response by TBS (*right panel*). **c** Averaged eEPSC amplitude of naive and TBS synapses (*left panel*) or another control group which experiences 1 h of LFS at 0.05 Hz without TBS (*right panel*). **d** Averaged eEPSC Ƭ_fast_ and Ƭ_slow_ values from naïve, TBS and LFS control synapses. **e**-**f** Plots summarizing time dependent changes in Ƭ_fast_ and amplitude of eEPSCs after TBS compared to naïve controls. There are significant differences (*p* < 0.05) in both parameters between 15 and 45 min in TBS group but not naïve control group. **g** Paired pulse ratio (PPR) at intervals of 3.3, 5 and 10 ms are plotted for naïve and TBS synapses. Holding potential was − 60 mV for this and subsequent figures. Statistical analysis is performed between neuron populations with unpaired t-tests with significance denoted as * *p* < 0.05

Slices were transferred to a perfused recording chamber mounted on a Zeiss Axioskop microscope with a 60x objective. The perfusion solution consisted of oxygenated aCSF with 2 mM CaCl_2_ and 1 mM MgCl_2_ supplemented with 10 μM bicuculline and 1 μM strychnine to block inhibitory inputs as well as 10 μM glycine to facilitate NMDAR activation. A bipolar stimulation electrode was placed near the midline of slices for stimulation of presynaptic axons. Stimulation voltage was set at 20% above the response threshold. In all cases described for these experiments, all-or-none responses were recorded from individual visually identifiable MNTB neurons. Cell-attached and whole-cell voltage clamp recordings were made from MNTB neurons with borosilicate glass electrodes pulled to a tip resistance of 2-3MΩ filled with an intracellular solution containing (in mM) 97.5 K-gluconate, 32.5 CsCl, 5 EGTA, 10 HEPES, 1 MgCl2, 30 TEA, and 3 QX314, pH 7.3. Series resistance for voltage-clamp recordings was 2–5 MΩ and compensated to 90% with a lag of 10 μs. The following stimulation paradigm was used (Fig. 1a): single action potentials were evoked at a frequency of 0.05 Hz for 10 min to establish a stable baseline prior to a 2-min theta burst stimulation (TBS; 4 pulse burst at 50 Hz, one burst per second for 120 s) followed by a designated time period (15–45 min) minutes of low frequency stimulation (LFS) at 0.05 Hz. Any cells that experienced spontaneous membrane rupture during the cell-attached recordings were rejected from analysis. Following this period, whole-cell recordings were then performed from the cell that experienced TBS and neighbouring connected cells with the same or lower stimulation threshold. Naive cells in the opposing MNTB nucleus experienced no TBS stimulation protocol and served as controls for the same slices. These in-slice controls help reduce relatively large variance of different experiments and facilitate paired comparisons of results from developing synapses in young mice.

### Data acquisition and analysis

Evoked EPSCs (eEPSCs) were recorded at − 60 mV and elicited in all-or-none manner (characteristic of one-to-one innervation pattern of the calyx of Held-MNTB synapse) by stimulating afferents with single, pair or train stimuli at different intervals as described in the text. Quantification of eEPSCs decay time constants involved fitting the average decay with a dual exponential function to provide fast and slow decay time constants in Clampfit.

$$ f(t)={A}_{fast}{e}^{\Big(-t/T{fast}\Big)}+{A}_{fast}{e}^{\Big(-t/{T}{slow}\Big)}+C $$

*Where A* is the relative amplitude of fast or slow component; *t* is time; Ƭ is decay time constant of fast or slow component; *C* is the convolution constant.

Miniature EPSCs (mEPSCs) were also recorded at -60 mV and fit individually with the identical double exponential decay function using MiniAnalysis software (Synaptosoft). Because Ƭ_fast_ values were mainly determined by synaptic AMPARs without contamination of NMDARs at -60 mV, only Ƭ_fast_ values from mEPSCs were binned for the generation of histograms (0.1 ms bin width) using Clampfit (Axon Instruments) and compared among different experimental conditions. To account for the variable number of events in each histogram, the resulting area of the distribution was normalized to 1 to allow for accurate pair wise comparisons. Histograms of mEPSC Ƭ_fast_ values from individual neurons were then fit with a double component Gaussian function:

$$ f(t)={\sum}_{i=1}^n Ai\frac{e^{-{\left(\tau fast-\mu i\right)}^2/2{\sigma}_i^2}}{\sigma_i\sqrt{2\pi }} $$

*Where A* is the relative area; *τ*_*fast*_ is time constant; μ is the mean of time constants; σ is time distribution standard deviation.

Quantification of the changes in mEPSC decay kinetics is reported as the relative area (*A*) of which each constituent Gaussian component comprises the entire distribution with two components being complementary.

### Immunohistochemistry

Two hundred to Two hundred fifty micrometer sections were acquired in the aforementioned manner. In order to label presynaptic terminals to facilitate their subsequent identification in fixed tissue, TBS was elicited through presynaptic current injection using a patch pipette (5–6 MΩ resistance) containing 0.5% Alexa555 labeled dextran (Invitrogen, #D-22910) in the intracellular solution which contained (in mM): 97.5 K-gluconate, 32.5 KCl, 0.5 or 10 EGTA, 40 HEPES, 1 MgCl_2_, and 3 K-glutamate, pH 7.3. Following the induction and expression period, which allowed for passive diffusion of the label into the terminal, pipettes were slowly removed to facilitate resealing of the plasma membrane. A neighbouring unconnected synapse was also labelled in a similar fashion to act as a naive control. Sections were then fixed for 30 min in cold 4% paraformaldehyde (PFA). PFA was rinsed from sections with 3 successive rinses of PBS. Cells were then permeablized with 30 min incubation in 0.2% triton-X 100 followed by another 3 rinses in PBS. Blocking was performed with 2 h incubation in 10% normal goat serum. Sections were then placed into primary antibody incubation (1:400 αGluA4, #AB1508 Millipore), prepared in the same blocking solution, overnight (approximately 14–18 h) with gentle agitation. Sections were then given 3 PBS rinses followed by 2 h incubation in Cy5 conjugated goat α rabbit (1:500 ThermoFisher Scientific, #A10523). From this stage on, all reactions took place in a dark room. Secondary antibody was then rinsed with 3 PBS washes and sections mounted on glass slides.

### Imaging

Images were acquired with a Zeiss LSM 510 Multiphoton Laser Scanning Microscope equipped with, 405, 488 and 514 nm argon laser lines. Confocal z-stack scans (0.5 μm steps) were acquired using a 63X (N.A. 1.4) oil immersion objective and the appropriate dichroic filters. 3D images were rendered and fluorescence intensity measurements were performed using Velocity software (Perkin Elmer). GluA4 staining intensity was reported as the mean intensity of Cy5 labeling in the region of the postsynaptic membrane immediately opposite the Alexa555 labeled terminal. To avoid bias, automated fluorescence detection was used to ensure only terminal adjacent regions of the postsynaptic membrane were analysed with results acquired by two individuals in a double-blind manner.

### Statistics

Since our experiments were performed in cell-attached configuration, the acquisition of pre-TBS mEPSCs in tetanized cells was not possible; therefore comparisons were made between cell populations. mEPSCs decay kinetics of stimulated synapses was therefore compared to naïve controls from the same slice (Fig. 1b). Using same slice naive controls for all experiments minimizes the presence of any potential inter-slice variability. All results were expressed as mean ± standard error (SEM), and statistical comparison of different experimental populations were performed using unpaired Student’s t-tests calculated using GraphPad Software with significance being denoted as *p* < 0.05.

## Results

### Activity-dependent induction but delayed expression of acceleration in the time course of eEPSCs

In the developing auditory system, prior to onset of hearing, spontaneous spike discharges, typically in the form of short high-frequency bursts separated by long quiescent gaps, have been observed in neurons of different nuclei [3, 4, 17, 18], implicating important roles of such patterned activity in the development of synaptic functions and plasticity. Consistent with this, we have previously demonstrated that afferent simulation with a TBS paradigm consisting of 1 Hz bursts (50 Hz × 60 ms) for 2 min can lead to a rapid downregulation of extra−/perisynaptic NMDARs and enhance the fidelity of neurotransmission at immature calyx of Held-MNTB synapses (*P* < 12) [12]. Surprisingly, neither the amplitude nor the time course of AMPAR-EPSCs was affected in the same synapses. Given that we conducted these experiments in the postsynaptic whole-cell configuration, which may perturb intracellular signaling, we sought to perform perforate patch recordings that would allow for same cell comparisons. However, it was too difficult to achieve low access resistance (i.e. < 10 MΩ) and maintain its stability for 1 hour in order to implement the full paradigm to discern the differences, if any, in the kinetics of AMPAR-EPSCs. As an alternative, we used the cell-attached recording mode to preserve the cell integrity, and examine AMPAR-EPSCs in whole-cell mode by rupturing the membrane at designated time intervals (Δt) after the same TBS (Fig. 1a). To this end, we first sealed onto postsynaptic neurons under voltage-clamp and tested whether low frequency stimulation (LFS, 0.05 HZ) of afferents with a bipolar stimulation electrode can reliably trigger single spikes in the form of extracellular compound action currents. Only those that responded in an all-or-none manner as a result of single axosomatic innervation of the postsynaptic neuron proceeded with the TBS paradigm (Fig. 1a). After TBS, we continued to monitor the connected synapse with LFS for various periods of time (15–45 min) before the membrane of the postsynaptic neurons were ruptured to establish the whole-cell configuration sequentially at different time points. Both eEPSCs and mEPSCs from tetanized synapses were recorded at a − 60 mV holding potential and compared to those from naïve synapses in contralateral MNTB. Figure 1b contrasts two sets of averaged eEPSC traces from naïve and tetanized synapses in the same slice 45 min after TBS, showing that the amplitude was reduced (Amplitude: 3.94 ± 0.25 nA, *n* = 23, vs. 3.06 ± 0.33 nA, *n* = 20, Degrees of Freedom (df) = 41, *p* = 0.04; Fig. 1c) and their time course was accelerated. When the decay phase of the mean eEPSC was fit with a dual exponential function, we found that fast and slow decay time constants (τ_fast_ and τ_slow_ respectively) of mean eEPSCs in tetanized synapses showed a reduction compared to that of naïve controls (τ_fast_ & τ_slow_: Naïve 1.43 ± 0.06 ms and 7.37 ± 0.62 ms, *n* = 23, vs. TBS 1.07 ± 0.06 ms and 5.34 ± 0.43 ms, *n* = 20; df = 41, *p* = 0.0002 for τ_fast_; df = 41, *p* = 0.0132 for τ_slow_; Fig. 1d). This acceleration in the decay kinetics and reduction in eEPSC amplitude was associated with a reduction of extrasynaptic NMDAR currents measured at + 60 mV (Naïve 4.35 ± 0.69 nA, *n* = 6, vs. TBS 3.25 ± 0.49 nA, *n*= 6), in line with what we previously reported [12].

To specifically test the role of the TBS, we also performed parallel control experiments in which LFS was continually delivered for 1 h prior to breakthrough in the absence of TBS. We found that there were minimal changes in eEPSC decay time constants or amplitude (Ƭ_fast_: Naive 1.39 ± 0.13 ms, *n*= 8 vs. LFS 1.36 ± 0.11 ms, *n*= 8, df = 14, *p*= 0.8627; Ƭ_slow_: Naive 7.98 ± 0.67 ms, *n*= 8 vs LFS 7.58 ± 1.44 ms, *n*= 8, df = 14, *p*= 0.8048; Amplitude: Naive 3.84 ± 0.48 nA, *n*= 8 vs. LFS 3.54 ± 0.48 nA, *n*= 8, df = 14, *p*= 0.6653; Fig. 1c, d). These results indicated that TBS can reliably and specifically induce plastic changes in the size and kinetics of eEPSCs.

To determine the time course of this TBS induced plasticity in eEPSCs, we sequentially ruptured the membrane at different time points following TBS (5 min interval for 15–45 min) in 6 subsets of experiments. Both the reduction in eEPSC amplitude and the acceleration in decay kinetics began to emerge following an expression phase greater than 30 min following TBS (Ƭ_fast_: Naive 1.54 ± 0.07 ms, vs. TBS at 35 min 1.14 ± 0.07 ms, *n*= 4, df = 6, *p*= 0.005; Amplitude: Naive 6.20 ± 0.93 nA, vs. TBS at 35 min 3.22 ± 0.37 nA, *n*= 4, df = 6, *p*= 0.024 Fig. 1f, g). This result provides insights into why activity-dependent plasticity in AMPAR-EPSCs was not previously observed after the same TBS paradigm applied in the whole-cell recording configuration [12]. Intracellular signaling may have been disrupted with this invasive recording mode to preclude the expression of the plasticity. Interestingly, we also observed an increase in the paired pulse ratio (PPR) of tetanized synapses at different time intervals (PPR at 5 ms: TBS 0.79 ± 0.05, *n*= 11, vs. Naive 0.96 ± 0.06, *n*= 12, df = 21, *p*= 0.043; Fig. 1g), suggesting a decrease in the release probability following TBS, which likely contributes to the reduction in eEPSC amplitude.

### Activity-dependent remodeling of synaptic AMPARs

**Fig. 2:**
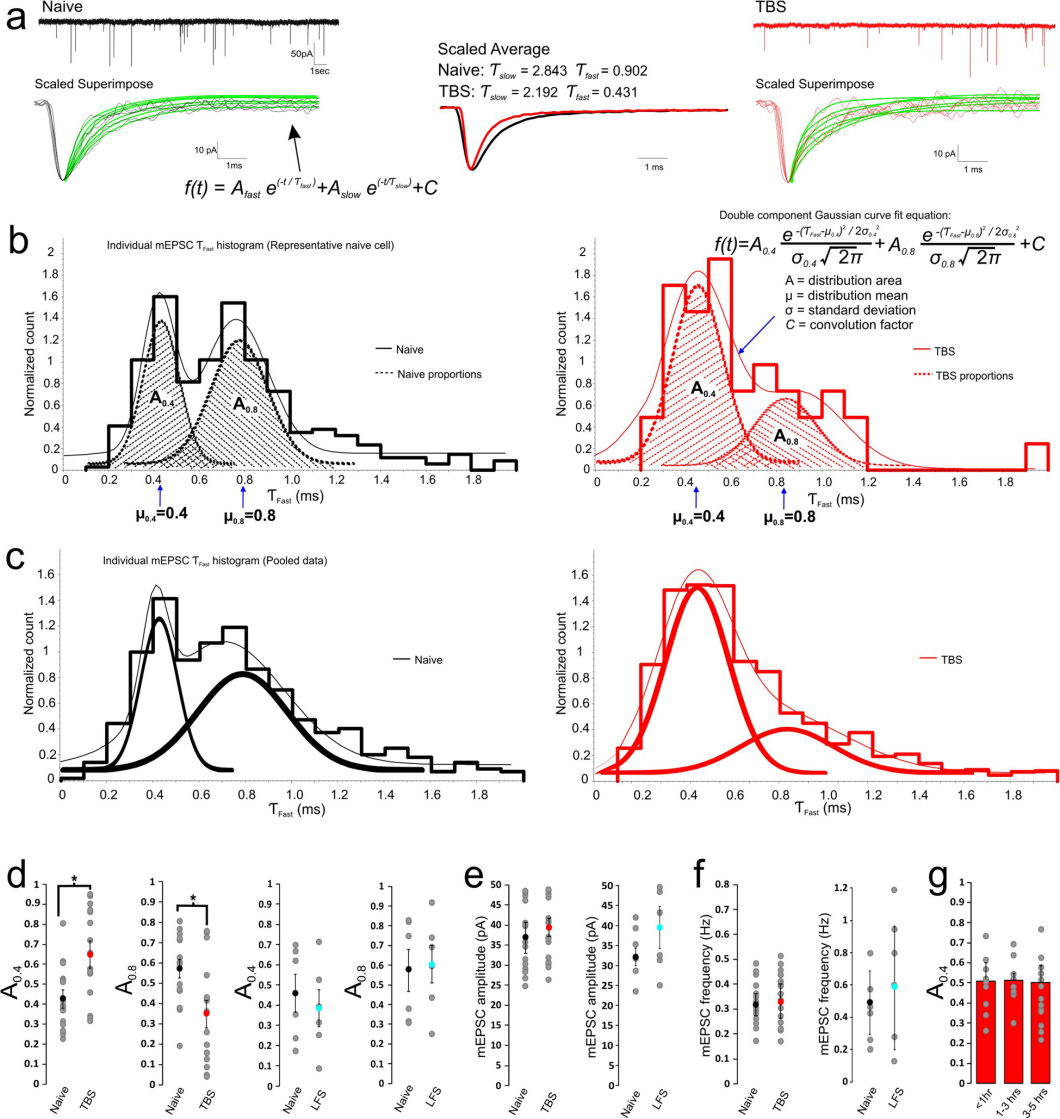
Activity-dependent redistribution of two cohorts of mEPSCs with distinct decay kinetics. **a** Representative mEPSC traces (*top panel*) from naive and TBS synapses are scaled and overlaid to show their variable decay time courses fit with a double exponential function (*bottom panel*). **b** τ_fast_ values from individual mEPSCs are plotted on conventional histograms with the total area under the curve being normalized to 1 and then fit with a double component Gaussian function to yield the relative weight of the fast (A_0.4_) and slow (A_0.8_) decay cohorts for naïve (*left panel*) and TBS synapses (*right panel*). **c** Comparison of naive and TBS treated synapses (pooled from all synapses in each group) exemplifies a decrease in the relative weight of the slow population (A_0.8_) and increase in the weight of the fast population (A_0.4_) following TBS. **d** The relative weights of the A_0.4_ and A_0.8_ cohorts from TBS and LFS treated synapses compared to their associated naive controls. **e**-**f** Averaged mEPSC amplitudes and frequency of TBS and LFS treated synapses with their associated naive controls. **g** Comparison of naïve synapses which spent varying amounts of time in recording solution

Since the change in PPR may implicate a presynaptic contribution to the plasticity in eEPSCs, we next examined the properties of mEPSCs which can be regarded as direct readouts of postsynaptic AMPARs in response to stochastic quantal release of glutamate from many release sites at the calyx of Held terminal. Figure 2a shows recordings of mEPSCs from naïve and tetanized synapses 45 min after TBS, in which individual mEPSCs were scaled and superimposed to show heterogeneity in their time courses. To quantitatively compare the kinetic differences, we fit the decay phase of individual single mEPSC events with a double exponential decay function (Fig. 2a), which yielded better fits than single exponential function. While it is commonly believed that NMDAR conductance is absent from mEPSCs, attributable to a voltage-dependant block by Mg^2+^, Espinosa and Kavalali [19] demonstrated that mEPSCs recorded at resting membrane potential, approximately 20% of the charge transfer is mediated by NMDARs. Given the high level of NMDAR expression in immature MNTB neurons and the inclusion of the NMDAR co-agonist glycine in the recording solution, contribution of NMDAR conductance to the later components of mEPSC decays might occur. As such, the τ_fast_ values of these double exponential fits, which comprise between 43 and 48% of the total fit weight, were used as the metric to gauge the decay kinetics of mEPSCs mediated by synaptic AMPARs. To rule out any confounding contribution of NMDARs to mEPSCs, we only used the τ_fast_ value for quantitative comparison under different experimental conditions throughout this study.

When all τ_fast_ values from a large number of mEPSCs were binned at 0.1 ms and plotted on a conventional histogram, we found their distribution patterns were multimodal, showing two primary cohorts which can be well described by dual-component Gaussian curves for both naïve and tetanized synapses, with the peak values centered around 0.4 ms (μ_0.4_) and 0.8 ms (μ_0.8_), respectively (Fig. 2b). However, the relative weight, quantified with their relative area (*A* values) in a double Gaussian fit (See Methods), of the first cohort increased while that of the second population decreased for the tetanized synapse compared to the naïve synapse in the same slice. When all data from the naïve control and tetanized synapses (15 synapses/group) were pooled, we found that the mean A_0.4_ was increased from 0.43 ± 0.05 in control to 0.65 ± 0.07 after TBS (df = 28, *p*= 0.0162), and the mean A_0.8_ was reduced in a complementary fashion from 0.57 ± 0.05 to 0.35 ± 0.07 (df = 28, *p*= 0.0162, total mEPSC events: Naive = 1446, TBS = 1219) (Fig. 2c, d). The significant shift in the relative weight of the 1st vs. the 2nd cohort of mEPSCs was not associated with marked changes in their amplitude (Naive 36.8 ± 3.9 pA, *n*= 15 vs. TBS 39.5 ± 2.3 pA, *n*= 15, df = 28, *p*= 0.56) and frequency (Naive 0.32 ± 0.05 Hz, *n*= 15, vs. TBS 0.33 ± 0.08 Hz, *n*= 15, df = 28, *p*= 0.916) (Fig. 2f). The observed changes were TBS dependent since in the LFS control group, amplitude, frequency and A values were similar to values in the corresponding naïve group (Fig. 2d, e). To determine that the acceleration in gating kinetics did not arise as a result of a time dependant shift in the distribution of mEPSC decays, naïve cells were assessed at varying time points after the immersion of slices into the recording bath. No differences were observed in the A_0.4_ proportions of cells which spent < 1 h, 1–3 h or 3-5 h in recording solution (Fig. 2g) (< 1 h: A_0.4_ = 0.51 ± 0.09, *n* = 9; 1–3 h A_0.4_ = 0.52 ± 0.03, *n* = 8; 3–5 h: A_0.4_ = 0.51 ± 0.07, *n* = 13; 1-way ANOVA F(2,27) = 0.479, *P* = 0.6247). These data suggest that there are two populations of mEPSCs in the developing calyx-MNTB synapse, with τ_fast_ values clustered around the distinct means of μ = 0.4 ms and μ = 0.8 ms, and that intense synaptic activity can increase the relative weight of the 1st cohort of mEPSCs at the expense of the 2nd cohort. Because mEPSCs read out stochastic quantal release from all presynaptic active zones, and the decay kinetics of mEPSCs is largely determined by deactivation of postsynaptic AMPARs present in a single postsynaptic density, we interpreted our observations as such that there are two primary homomeric populations of native synaptic AMPARs in the early developmental stage. The redistribution of the two populations suggests that synaptic AMPARs can undergo a subunit switch from slow-gating to fast-gating AMPARs after intensive synaptic activity, and contribute to the overall acceleration in the time course of eEPSCs. Given that mEPSC amplitude and frequency remain unaltered by TBS, we suggest that the reduction in the amplitude of eEPSCs must be mediated by presynaptic mechanisms that impact spike-dependent synchronized glutamate release, as also implicated by PPR of eEPSCs (Fig. 1f, g).

### Activation of NMDARs and Group1 mGluRs are required for gating switchof synaptic AMPARs

**Fig. 3:**
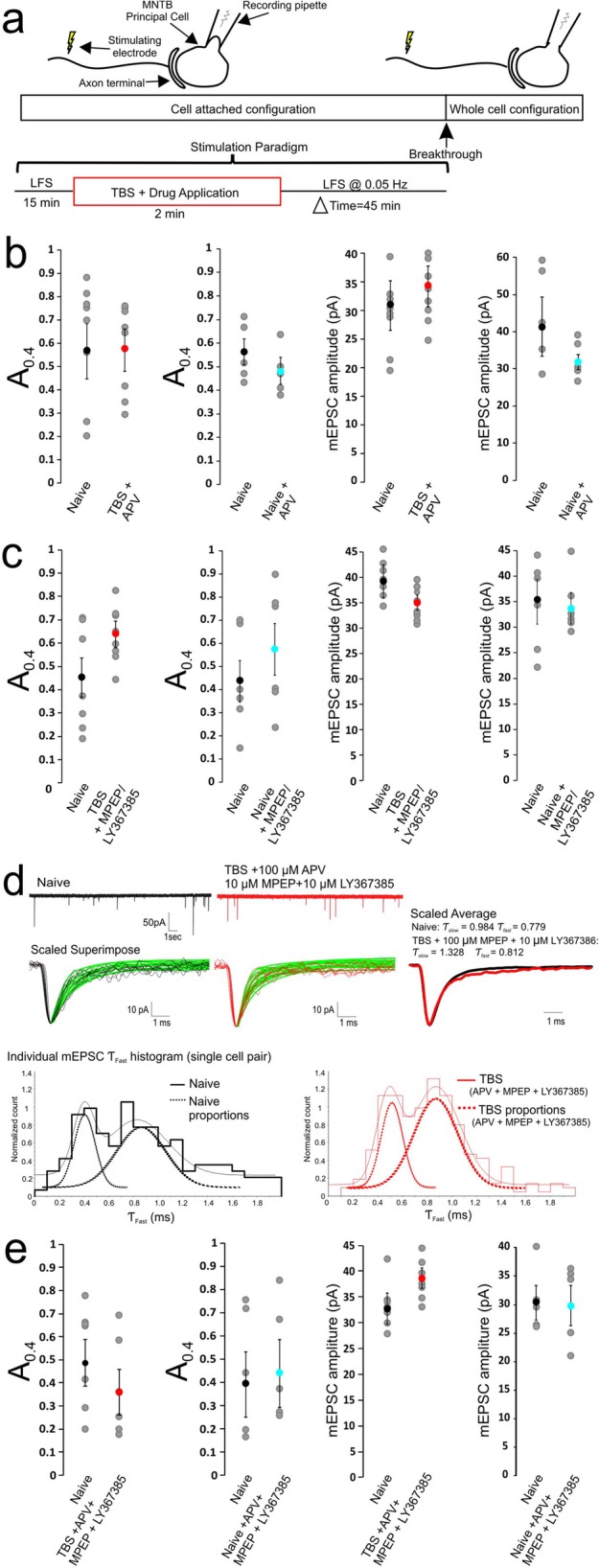
Blockade of NMDARs or Group 1 mGluRs prevents synaptic AMPAR gating switch. **a** The same experimental paradigm as in Fig. 1a except that NMDAR blocker (100 μM APV) and/or Group 1 mGluR blockers (10 μM MPEP + 10 μM LY367385) was applied during TBS. B-E). Summary plots showing APV (**b**) or MPEP+LY367385 (**c**) or in combination (**d**, **e**) blocks TBS induced increase in the relative size of A_0.4_ while the amplitude of mEPSCs is not affected in all conditions

We have previously demonstrated that TBS can lead to concurrent activation of NMDARs and Group 1 mGluRs and drive the down-regulation of peri−/extrasynaptic NMDARs [12]. Given that the same TBS paradigm induces a delayed gating switch in mEPSCs, we hypothesized that NMDARs and Group I mGluRs are required for the induction. To test this, we applied pharmacological antagonists for these 2 receptor classes alone or in combination only during the 2 min TBS (Fig. 3a). We found that the NMDAR antagonist 100 μM APV ((2*R*)-amino-5-phosphonopentanoate) prevented both the reduction in amplitude and acceleration of their decay. Distribution histograms of τ_fast_ values for mEPSCs remained multimodal, with all parameters remaining unchanged between naive and test populations (Naïve: A_0.4_ = 0.53 ± 0.12, A_0.8_ = 0.47 ± 0.12, *n*= 6 vs. APV + TBS: A_0.4_ = 0.55 ± 0.09, A_0.8_ = 0.45 ± 0.09, *n*= 6), while the mean amplitude of mEPSCs was not different either (Fig. 3b, Table 1A). In a separate control group to rule out any confounding effects of the drug application, we applied APV alone to naïve synapses without TBS, and found it had little effect on the relative weight of two mEPSC populations or their amplitude (Naïve: A_0.4_ = 0.56 ± 0.05, A_0.8_ = 0.44 ± 0.05, *n* = 5 vs. Naïve+APV: A_0.4_ = 0.49 ± 0.06, A_0.8_ = 0.51 ± 0.06, *n* = 5) (Fig. 3b, Table 1). These results suggest that the activation of NMDARs during the TBS is required to induce the gating switch.

**Table 1:**
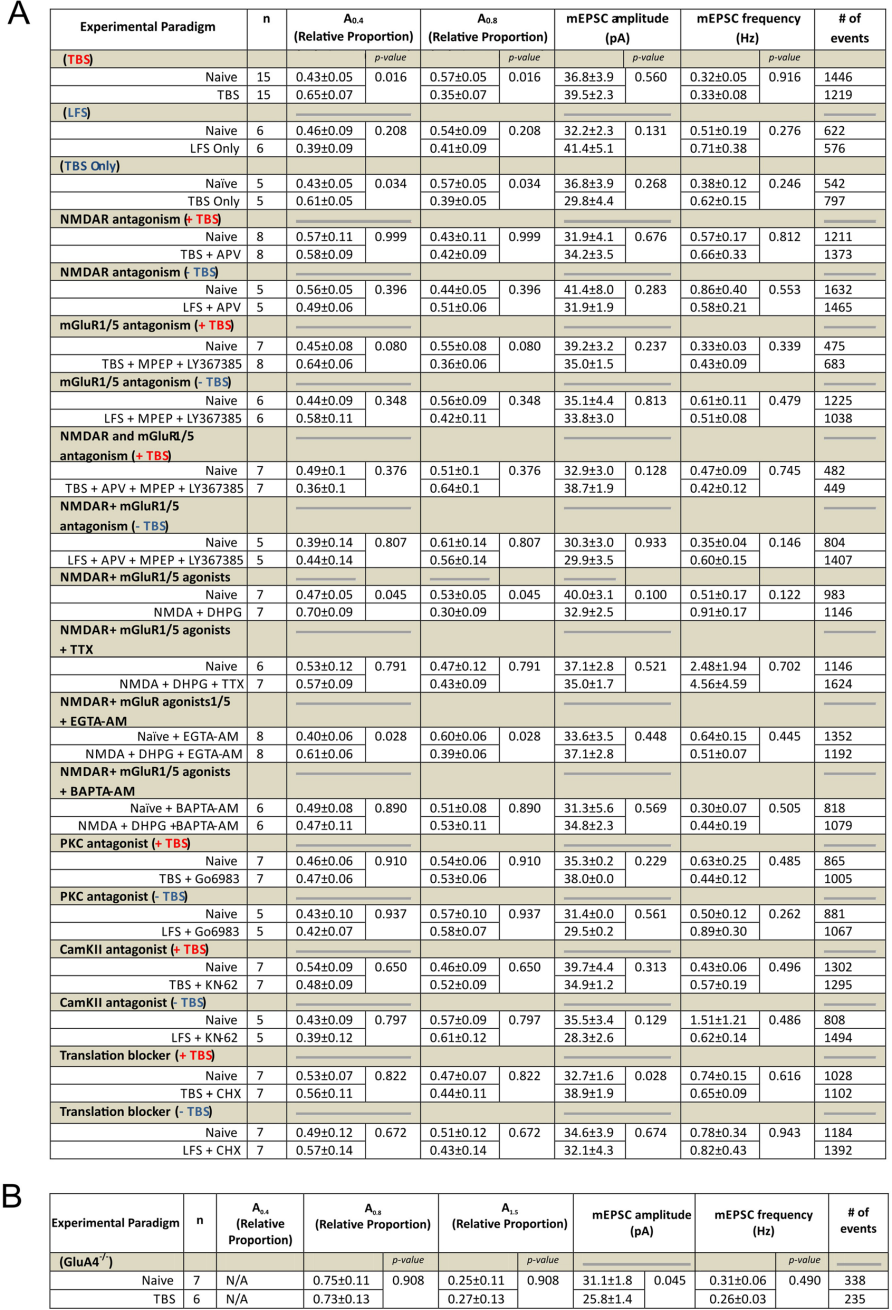
Key mEPSC parameters associated with activity-dependent AMPAR plasticity for wild-type (A) and GluA4^−/−^ synapses (B) Statistical comparison utilize unpaired Student’s t tests with significance being denoted as *p* < 0.05

We next tested the effects of antagonists of Group 1 mGluRs 10 μM MPEP [2-Methyl-6-(phenylethynyl)pyridine] for mGluR5 and 10 μM LY367385 for mGluR1. When we applied these antagonists during the TBS, we again found that TBS-induced acceleration in the decay time course of both eEPSCs (data not shown) and mEPSCs was prevented (Naïve: A_0.4_ = 0.45 ± 0.08, A_0.8_ = 0.55 ± 0.08, *n*= 7 vs. MPEP+LY367385 + TBS: A_0.4_ = 0.64 ± 0.06, A_0.8_ = 0.36 ± 0.06, *n*= 8) while mEPSC amplitude remained unchanged (Fig. 3c, Table 1A). Brief application of mGluR blockers alone without TBS had no effect on the relative weight of fast and slow populations of mEPSCs (Naïve: A_0.4_ = 0.44 ± 0.09, A_0.8_ = 0.56 ± 0.09, *n*= 6 vs. Naïve + MPEP + LY367385: A_0.4_ = 0.58 ± 0.11, A_0.8_ = 0.42 ± 0.11, *n*= 6) (Fig. 3c, Table 1A). These results suggest that Group 1 mGluRs were also required for induction of the acceleration in the kinetics of synaptic AMPARs. As expected, no significant changes in the amplitude and kinetics of mEPSCs were noted when both NMDARs and Group 1 mGluRs were blocked with a combination of APV, MPEP and LY367385 during TBS, as exemplified in recordings of mEPSCs with the accompanying histograms (Fig. 3d). Pooled data from two groups were summarized and compared (Naïve: A_0.4_ = 0.49 ± 0.10, A_0.8_ = 0.51 ± 0.10, *n* = 7 vs. APV + MPEP+LY367385 + TBS: A_0.4_ = 0.36 ± 0.10, A_0.8_ = 0.64 ± 0.10, *n* = 7) (Fig. 3e, Table 1). These antagonists did not exert significant effects on the properties of mEPSCs of naïve synapses in the absence of TBS (Naïve: A_0.4_ = 0.39 ± 0.14, A_0.8_ = 0.61 ± 0.14, *n* = 5 vs. Naïve+APV + MPEP+LY367385: A_0.4_ = 0.44 ± 0.14, A_0.8_ = 0.56 ± 0.14, *n* = 5) (Fig. 3e, Table 1A). Taken together, these results suggest that blocking either NMDAR or Group I mGluR signaling can preclude the delayed gating switch in synaptic AMPARs, raising the possibility that these two classes of receptors during TBS are both engaged in the induction of activity-dependent plasticity in AMPARs, similar to the induction requirements for the previously described down-regulation of peri−/extrasynaptic NMDARs.

**Fig. 4:**
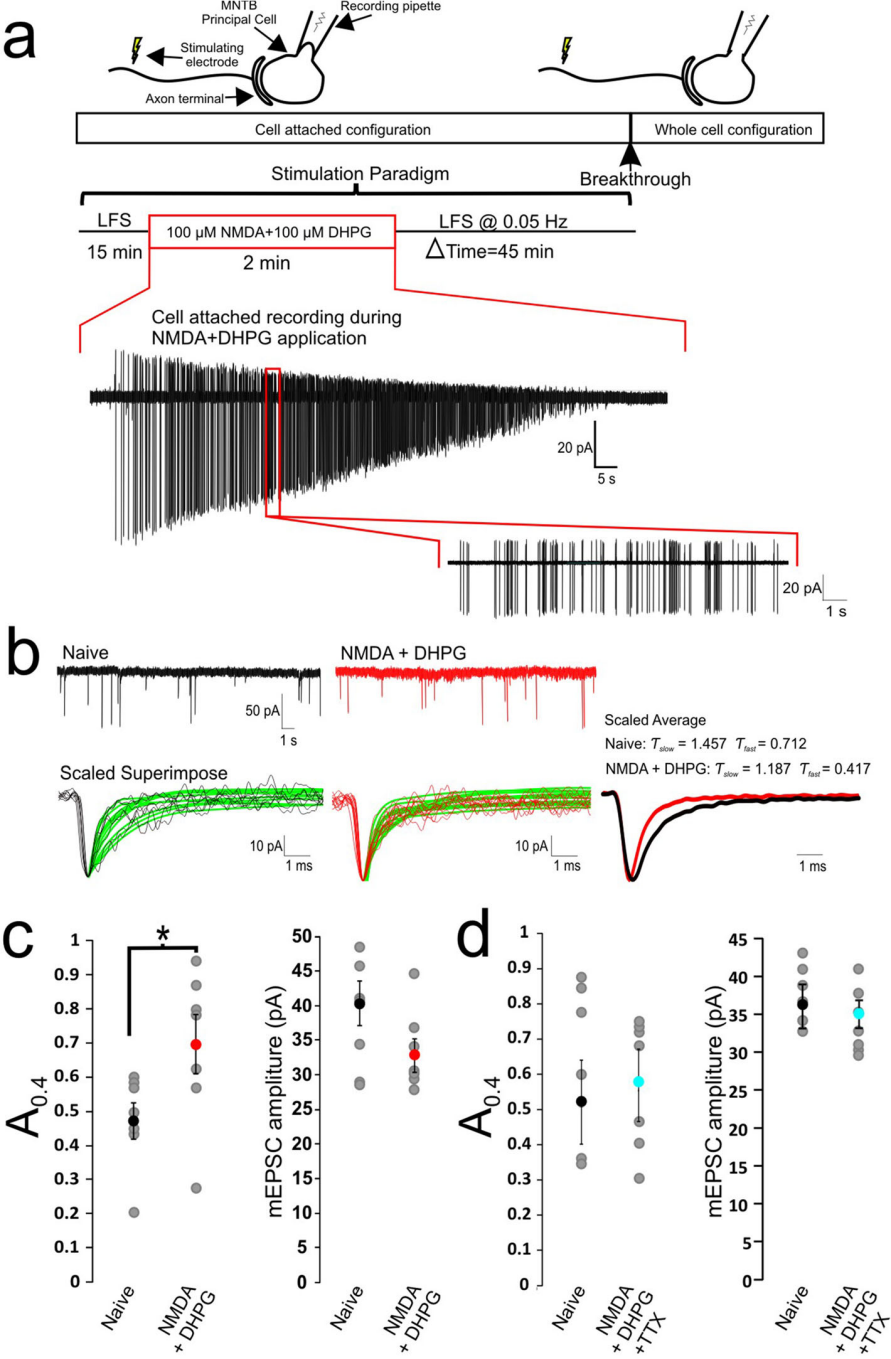
The gating switch of synaptic AMPARs can be pharmacologically recapitulated. **a** The same experimental paradigm except that TBS is replaced by a 2 min co-application of 100 μM NMDA + 100 μM DHPG, which evokes bursts of action potentials (30–70 Hz, 30–60 ms bursts, every 0.5–1 s). **b** Examples of raw mEPSCs (*top panels*) are scaled and superimposed with accompanying curve fits (*middle panels*) for naïve (*left column*) and drug treated synapses (*right column*). The averaged mEPSCs are contrasted to show acceleration in the time course of mEPSCs after co-activation of NMDARs and Group 1 mGluRs after a 45 min non-invasive expression phase. **c**. Summary plots showing an increase in the size of A_0.4_ component of mEPSCs without affecting mEPSC amplitudes after co-activation of NMDARs and mGluRs. **d** Blocking action potentials with 1 μM TTX during co-application of the agonists prevents the increase in the relative weight of A_0.4_ component

To further test that idea that activation of NMDARs and Group I mGluRs is important for the induction of the delayed gating switch, we directly co-applied receptor agonists (100 μM NMDA and 100 μM DHPG (− 3,5-Dihydroxyphenylglycine)) for a 2 min period in the absence of electrical stimulation to afferents. During cell-attached recordings, this application evoked 30–70 Hz bursts of action potentials (registered as compound inward and outward currents under voltage-clamp mode) at a frequency between 1 and 2 Hz (Fig. 4a), resembling the firing patterns evoked by the TBS paradigm through afferent stimulation as described in previous experiments. After cell-attached recording for 45 min, we ruptured the membrane and recorded mEPSCs, which were analysed post hoc with exponential fits to each event as described previously (Fig. 4b). The histogram of τ_fast_ values showed a similar multimodal distribution pattern, with NMDA/DHPG treated synapses showing a significant increase in the relative weight of the 1st cohort of mEPSCs over the 2nd one, compared to naïve control synapses with no significant change in mEPSC amplitude (Naïve: A_0.4_ = 0.47 ± 0.05, A_0.8_ = 0.53 ± 0.05, *n* = 7 vs. NMDA = DHPG: A_0.4_ = 0.70 ± 0.09, A_0.8_ = 0.30 ± 0.09, *n* = 7) (Fig. 4b, c, Table 1A). Neither NMDA nor DHPG alone was sufficient to induce a similar shift in their relative weight. These results demonstrated that activation of postsynaptic NMDARs and Group I mGluRs can directly trigger theta burst firings of postsynaptic neurons to induce a gating switch in synaptic AMPARs. Such burst firings were evidently important because co-application of 1 μM TTX blocked action potentials generated by NMDA and DHPG, and consequentially mEPSCs showed no switch in the relative weight of fast and slow mEPSCs, nor any changes in amplitude (Naïve: A_0.4_ = 0.49 ± 0.12, A_0.8_ = 0.51 ± 0.12, *n* = 5 vs. NMDA+DHPG+TTX: A_0.4_ = 0.55 ± 0.10, A_0.8_ = 0.45 ± 0.10, *n* = 6) (Fig. 4d, Table 1A). These results implicate that postsynaptic burst firings may play a synergistic role in amplifying NMDAR- and mGluR-dependent signaling for the induction of the activity-dependent gating switch of synaptic AMPARs.

### Activity-dependent remodeling of synaptic AMPARs depends on the elevation of intracellular Ca^2+^

**Fig. 5:**
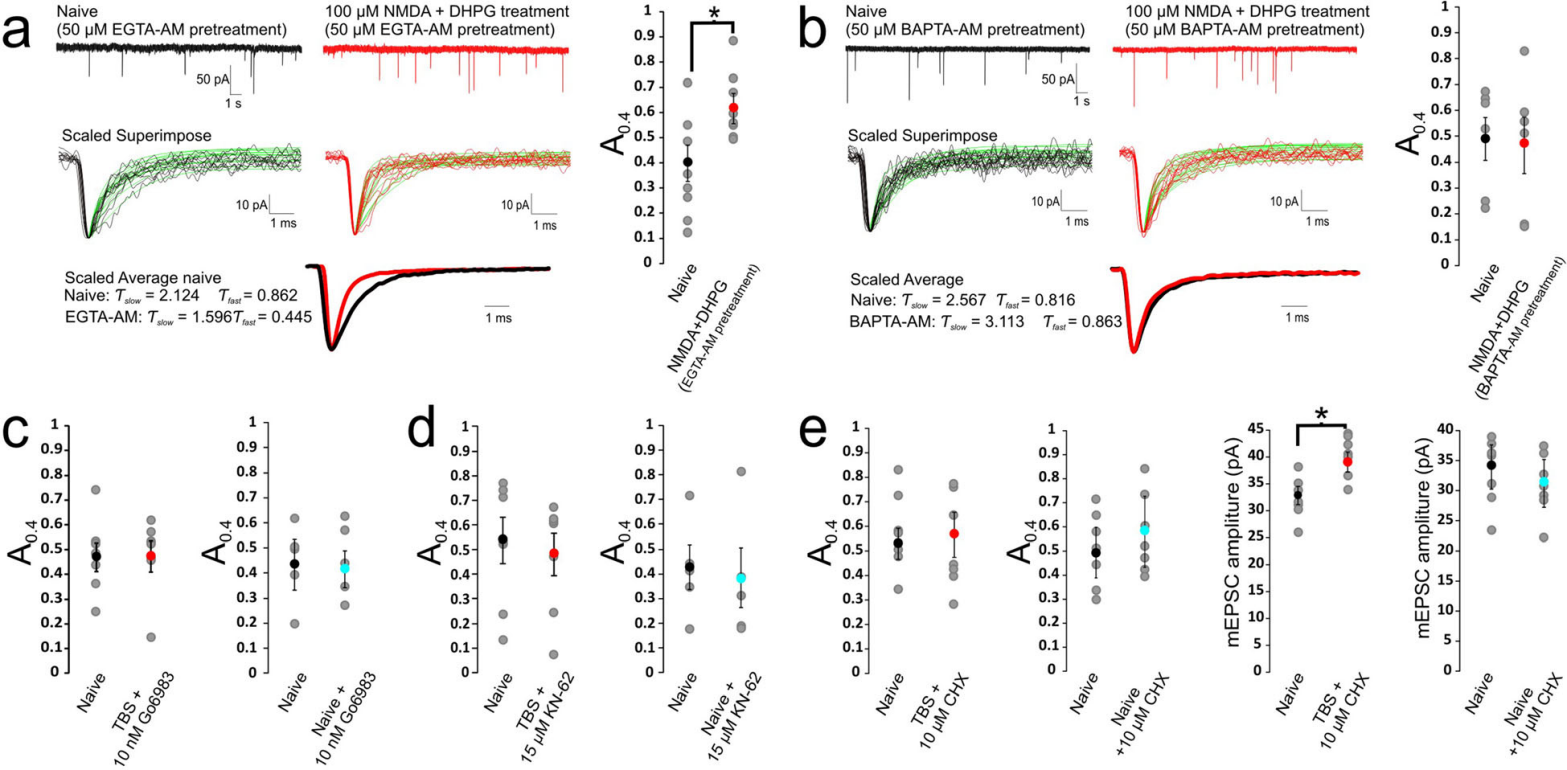
The AMPAR gating switch requires synergistic Ca^2+^ signaling to Ca^2+^ dependent kinases and protein synthesis. **a**-**b** Example recordings of raw and scaled mEPSCs from slices pretreated with EGTA-AM (**a**) or BAPTA-AM (**b**) followed by co-application of 100 μM NMDA + 100 μM DHPG show that BAPTA but not EGTA blocks the increase in the size of A_0.4_ of mEPSCs. **c-e** Summary plots showing PKC inhibitor (10 nM Go6983) (**c**) or CamKII blocker (15 μM KN-62) (**d**) or protein synthesis inhibitor (10 μM cyclohexamide) (**e**) blocks TBS-induced increase in the size of A_0.4_ component of mEPSCs. Note that cycloheximide increased the amplitude of mEPSCs in TBS treated synapses but not naïve synapses

During the early stage of development at the calyx-MNTB synapse, high levels of NMDARs would generate a significant influx of Ca^2+^ into the postsynaptic cell during trains of high frequency stimulation [7, 20]. Since postsynaptic action potentials are required to fully relieve the block of NMDARs by endogenous Mg^2+^ to allow Ca^2+^ influx while co-activation of Group 1 mGluRs is commonly associated with intracellular Ca^2+^ release through its coupling to IP3 receptors [21, 22], we reasoned that extracellular Ca^2+^ influx via NMDARs must be coupled to mGluR-dependent Ca^2+^ release for the induction of activity-dependent plasticity in synaptic AMPARs. To test this, neurons were incubated in 50 μM EGTA-AM to buffer cytosolic Ca^2+^ rise followed by co-application of NMDA and DHPG. This pharmacological induction approach was employed to avoid confounding presynaptic effects of EGTA in immature synapses [23, 24]. Surprisingly, synapses pretreated with EGTA-AM still demonstrate a bimodal redistribution in τ_fast_ values of mEPSCs with a significant increase in the component of the 1st cohort of mEPSCs following NMDA/DHPG treatment (Naïve: A_0.4_ = 0.41 ± 0.07, A_0.8_ = 0.59 ± 0.07, *n* = 7 vs. NMDA+DHPG+EGTA-AM: A_0.4_ = 0.63 ± 0.06, A_0.8_ = 0.37 ± 0.06, *n* = 7) (Fig. 5a, Table 1A). In contrast, recordings from slices that were pre-incubated in the fast Ca^2+^ buffer BAPTA-AM (50 μM) showed that co-application of NMDA and DHPG failed to induce the gating switch (Naïve: A_0.4_ = 0.49 ± 0.08, A_0.8_ = 0.51 ± 0.08, *n* = 6 vs. NMDA+DHPG+BAPTA-AM: A_0.4_ = 0.47 ± 0.11, A_0.8_ = 0.53 ± 0.11, *n* = 6) (Fig. 5b, Table 1A). Knowing that EGTA has much slower forward binding rate to capture Ca^2+^ than BAPTA, we suggest that there must be a tight spatial nanodomain coupling between Ca^2+^ influx from NMDARs and Ca^2+^ release from internal stores mediated by Group1 mGluRs for the induction of the gating switch.

### Ca^2+^-dependent protein kinases and protein synthesis are required for remodeling of synaptic AMPARs

Downstream of the activity-dependent Ca^2+^ rise in postsynaptic neurons, Ca^2+^-dependent protein kinases, most notably PKC and CaMKII, are well established to be associated with phosphorylation and trafficking of AMPARs underlying synaptic plasticity [25, 26]. To test the involvement of these Ca^2+^ sensitive kinases in the AMPAR gating switch at the calyx of Held-MNTB synapse, we first applied a broad-spectrum PKC inhibitor Go6789 (10 nM) during TBS, and found a complete blockade of redistribution of τ_fast_ values for the 1st and 2nd cohort of mEPSCs (Naïve: A_0.4_ = 0.46 ± 0.06, A_0.8_ = 0.54 ± 0.06, *n* = 7 vs. Go6983 + TBS: A_0.4_ = 0.47 ± 0.06, A_0.8_ = 0.53 ± 0.06, *n* = 7); PKC inhibitor itself did not have any effect on naïve synapses either (Naïve: A_0.4_ = 0.43 ± 0.10, A_0.8_ = 0.57 ± 0.10, *n* = 5 vs. Naïve = Go6983: A_0.4_ = 0.42 ± 0.07, A_0.8_ = 0.58 ± 0.07, *n* = 5) (Fig. 5c, Table 1A). Similarly, application of specific CaMKII antagonist, KN-62 (15 μM) during the TBS also blocked the AMPAR gating switch (Naïve: A_0.4_ = 0.54 ± 0.09, A_0.8_ = 0.46 ± 0.09, *n* = 7 vs. KN-62 + TBS: A_0.4_ = 0.48 ± 0.09, A_0.8_ = 0.52 ± 0.09, *n* = 7), while KN-62 application alone had no effect (Naïve: A_0.4_ = 0.43 ± 0.09, A_0.8_ = 0.57 ± 0.09, *n* = 5 vs. Naive+KN-62: A_0.4_ = 0.39 ± 0.12, A_0.8_ = 0.61 ± 0.12, *n* = 5) (Fig. 5d, Table 1A). These results indicate that these two kinases likely mediate the expression phase of this activity-dependent plasticity through phosphorylation-dependent reorganization of postsynaptic AMPARs.

Given the extended expression phase (> 30 min) is required for the expression of TBS-dependent redistribution of τ_fast_ values, it is plausible that protein synthesis plays a role in the gating switch. Proteins involved in forming endocytotic complexes, namely Arc/Arg3.1, are known to undergo rapid translational up-regulation within 1 h of Group 1 mGluR activation [27]. To test this, we exposed slices to the translation inhibitor cycloheximide (CHX, 10 μM) for 30 min following TBS, and found that this treatment effectively arrested the redistribution of in τ_fast_ values for two populations of mEPSCs (Naïve: A_0.4_ = 0.51 ± 0.06, A_0.8_ = 0.49 ± 0.06, *n* = 6 vs. CHX + TBS: A_0.4_ = 0.57 ± 0.09, A_0.8_ = 0.43 ± 0.09, *n* = 6) (Fig. 5e, Table 1A). However, while application of the translation inhibitor in the absence of TBS resulted in no changes to the proportion of the two decay populations (Naïve: A_0.4_ = 0.50 ± 0.10, A_0.8_ = 0.50 ± 0.10, *n* = 6 vs. Naïve+CHX: A_0.4_ = 0.56 ± 0.15, A_0.8_ = 0.44 ± 0.15, *n* = 6), the amplitude of mEPSCs showed a significant increase after CHX treatment (Fig. 5e, Table 1A). The control group of synapses which did not experience TBS, CHX itself demonstrated no effect on the amplitude of mEPSCs (Fig. 5e, Table 1A), implying the removal of AMPARs requires protein synthesis while insertion of AMPARs likely occurs via a translation independent mechanism to increase the amplitude of mEPSCs even in the presence of CHX. This is in agreement with a large body of work implicating rapid protein translation as a modulator of both synaptic plasticity and the maintenance of basal synaptic strength [28]. The absence of any acceleration in mEPSC decay kinetics may stem from a lack of removal of slow gating AMPARs and/or the inability of any newly recruited fast-gating AMPARs to effectively integrate into the post synaptic densities (PSDs) due to space constraints of postsynaptic density. These results collectively suggest that the TBS paradigm activates Ca^2+^-dependent post-translational modifications and protein synthesis, leading to a reorganization of native AMPARs from slow-gating to fast-gating phenotypes.

### Recruitment of GluA4 is critical for activity-dependent acceleration of eEPSCs

Previous work by our group and others has shown that the developmental acceleration of EPSCs at this synapse is largely mediated by a subunit switch in Ca^2+^ permeable GluA subtypes (i.e. inwardly rectifying current-voltage relationship in spermine throughout development) [7, 8]. Prior to the onset of hearing, synaptic AMPAR composition is dominated by GluA1 channels and a virtual absence of GluA2 subunits, as revealed by whole-cell and outside-out patch clamp recordings, immunohistochemistry and single cell RT-PCR of GluA transcripts [8, 9]. As synapses mature, fast gating GluA4 channels become more prominent with minimal contribution of GluA3, paralleled by the downregulation of slower gating GluA1 containing AMPARs [7–9, 11]. This gating switch is indispensable for driving fast neurotransmission at this synapse as demonstrated by studies in GluA4^−/−^ mice [11].

**Fig. 6:**
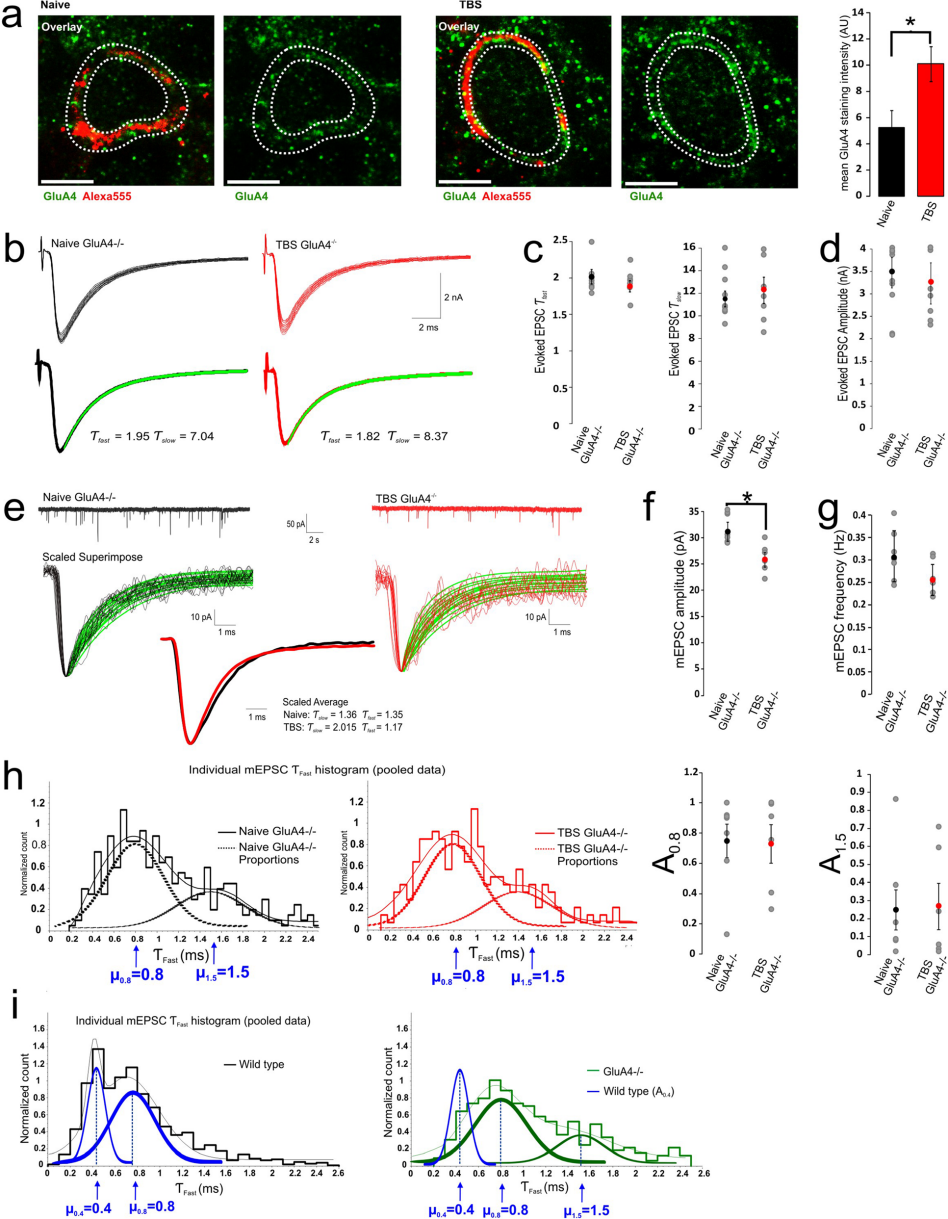
GluA4 is the molecular substrate mediating the acceleration in gating kinetics of eEPSCs and mEPSCs. **a** Examples showing immunofluorescent double-labeling of presynaptic terminal with tracer Alexa 555 (*Red*) and GluA4 antibody (*Green*) in synapses with or without TBS treatment. The same TBS paradigm as in Fig. 1 was delivered directly through presynaptic current injections (0.5–1 nA, 0.2 ms) with pipettes containing Alexa555 labeled dextran to allow post hoc identification of stimulated synapses in fixed slices (*n* = 4 slices from 4 mice). Naïve synapses were only injected with Alexa 555 but devoid of TBS paradigm as control. Average GluA4 membrane staining intensity was measured for the entire postsynaptic cell (representative z-stack section with the GluA4 intensity measured between white hatched lines). Scale bars: 10 μm for all panels. **b** Overlay of raw (*top panels*) and averaged eEPSCs (*bottom panels*) from naive (*left column*) and TBS treated synapses (*right column*) from GluA4^−/−^ mice with the accompanying dual exponential curve fits to the averaged traces and time constants given. **c**-**d** Summary plots of averaged Ƭ_fast_ and Ƭ_slow_ values of eEPSCs as well as the amplitude from naive and TBS synapses. **e** Raw and scaled mEPSCs from naive and TBS treated GluA4^−/−^ synapses are shown. **f-g** Plots of averaged mEPSC amplitudes and frequency from naive and TBS treated GluA4^−/−^ synapses. **h** Individual Ƭ_fast_ values of mEPSCs from pooled naive GluA4^−/−^ and TBS GluA4^−/−^ populations plotted on a conventional histogram normalized for area under the curve, showing no change in the relative proportions of two cohorts of mEPSCs (μ_0.8_ and μ_1.5_). **i** Comparison of Ƭ_fast_ histograms of mEPSCs from WT naive and GluA4^−/−^ naive synapses reveals the μ_0.4_ cohort in WT synapses is absent in GluA4^−/−^synapses

Gaussian fits to the multimodal histograms of τ_fast_ values of mEPSCs typically yield two distinct populations with μ_0.4_ = 0.4 ms and μ_0.8_ = 0.8 ms as shown previously. These values bear a striking similarity to the deactivation time constants of homomeric GluA4 and GluA1 AMPARs. Because synaptic AMPARs undergo a developmental transformation in composition from GluA1 to GluA4 dominant phenotype within the first two postnatal weeks, we postulate that the observed TBS induced acceleration of decay time course of mEPSCs may be indicative of an increase in the level of GluA4 subunits coupled with the removal of GluA1 in acute slices within a 1 h time span after the induction paradigm. We sought to use immunofluorescence to demonstrate that TBS could induce an increase in the GluA4 membrane staining intensity. To this end, we made whole-cell recordings from single calyx of Held terminals, in which the same TBS stimuli were delivered directly through current injections using presynaptic pipettes filled with the fluorescent indicator Alexa555. At the end of each recording, we slowly removed the pipette from the terminal to allow membrane reseal and constrain dye leakage, and immediately fixed slices for immunohistochemical experiments to quantitatively compare the levels of GluA4 with or without TBS as exemplified in two synapses in Fig. 6a. When relative GluA4 fluorescence intensities in the postsynaptic membranes directly adjacent to the A555 labelled terminals were compared, we noted that synapses which had undergone the TBS protocol showed a significant increase in GluA4 fluorescence intensity (10.11 AU ± 1.32, *n* = 4, *p* = 0.039) as compared to the controls without TBS (5.26 AU ± 1.29, *n* = 4)(Fig. 6a). This result suggests that there is an increase of GluA4 in the postsynaptic soma, and that GluA4 is likely the substrate mediating the acceleration in eEPSCs and mEPSC decay kinetics.

As an alternative method of validating an increase in GluA4 expression as the molecular substrate underlying the gating switch, we resorted to the electrophysiological characterization of GluA4 knockout mice because of a lack of subtype specific antagonists of AMPARs. We tested if the distribution pattern of τ_fast_ values in GluA4^−/−^ mice were different from that of wild-type, and if TBS remained effective in accelerating the time course of eEPSCs and mEPSCs. When compared to the decay time courses of eEPSCs from wild-type synapses (Fig. 1), we noted that eEPSCs from GluA4^−/−^ synapses have a much slower time course with τ_Fast_ and τ_slow_ exceeding 2 and 10 ms respectively (Fig. 6). When the same TBS paradigm was applied in cell-attached mode, we found no significant changes in their decay time constants (τ_Fast_: Naive GluA4^−/−^ 2.02 ± 0.10 ms, *n* = 7, vs. TBS GluA4^−/−^ 1.89 ± 0.08 ms, *n* = 5, df = 10, *p* = 0.37; τ_slow_: Naive Glu4^−/−^ 11.50 ± 0.69 ms, *n* = 7, vs. TBS GluA4^−/−^ 12.26 ± 1.16 ms, *n* = 5, df = 10, *p* = 0.56 Fig. 6b, c) or amplitude (Naive GluA4^−/−^ 3.47 ± 0.34 nA, *n* = 7, vs. GluA4^−/−^ TBS 3.23 ± 0.46 nA, *n* = 5, *p* = 0.68 Fig. 6d).

When we analyzed and compared the kinetics of individual mEPSCs from naïve GluA4^−/−^ and TBS treated GluA4^−/−^ synapses, we found that the distribution pattern of τ_fast_ values remained bimodal, but most notably the population with μ = 0.4 ms, as typically seen in wild-type synapses, disappeared. TBS induced no redistribution of two GluA4^−/−^ populations with μ = 0.8 and μ = 1.5 ms (A_0.8_: Naive GluA4^−/−^ 0.75 ± 0.11, *n* = 7, vs. GluA4^−/−^ TBS 0.73 ± 0.13, *n* = 6; A_1.5_: Naive GluA4^−/−^ 0.25 ± 0.11, *n* = 7, vs. GluA4^−/−^ TBS 0.27 ± 0.13, *n* = 6, df = 10, *p* = 0.91; Table 1B, Fig. 6e, h, i). The amplitude of mEPSCs from tetanized GluA4^−/−^ synapses however showed a decrease as compared to the naive control population (Naive GluA4^−/−^ 31.1 ± 1.78 pA, *n* = 7, vs. GluA4^−/−^ TBS 25.8 ± 1.40 pA, *n* = 6, df = 10, *p* = 0.045; Table 1B, Fig. 6f) while mEPSC frequency remained unchanged (Naive GluA4^−/−^ 0.31 ± 0.06 Hz, *n* = 6, vs. 0.26 ± 0.03 Hz, *n* = 6, df = 10, *p* = 0.47; Table 1B, Fig. 6g). We interpret these observations as such that there is an independent regulation of GluA1 removal regardless of absence or presence of GluA4. In other words, TBS has very little effect on the redistribution of τ_Fast_ populations in GluA4^−/−^ synapses but is capable in inducing a reduction in postsynaptic AMPARs, presumably GluA1 receptors. There is a minor component being at 1.4 ms in WT synapses, which is up-regualted in GluA4^−/−^ likely due to misalignment of AMPARs to release sites or a compensation by other GluAs at synaptic or perisynaptic sites [11]. We therefore conclude that GluA4 is not only the molecular correlate underlying the population of fast mEPSCs (A_0.4_), but also the substrate for activity-dependent AMPAR recruitment to the PSDs, necessary for the development of ultra-fast neurotransmission at the calyx of Held-MNTB synapse.

## Discussion

Our findings represent a new form of activity-dependent synaptic plasticity at a developing central synapse, in which a switch of the subunit composition of synaptic AMPARs from GluA1 to GluA4-dominant subtypes is induced by activity but with an unusual delay in its temporal onset of expression. By applying a condensed series of theta bursts to afferents of MNTB neurons in slices from prehearing mice, we induced the acceleration in the decay kinetics of evoked and miniature EPSCs only when the membrane integrity is maintained for a period exceeding 30 min. Using a combination of multimodal analyses of time constants from mEPSCs in WT and GluA4^−/−^, we further demonstrated that coincident activation of NMDAR and Group 1 mGluRs leads to Ca^2+^-dependent post-translational regulation and protein synthesis to recruit GluA4 dominant AMPARs into synapses at the expense of GluA1 dominant AMPARs. This acutely inducible form of activity-dependent plasticity in vitro closely models developmental plasticity in synaptic AMPARs in the first two postnatal weeks in vivo [5–10], providing an important platform to address molecular substrates underlying AMPAR mediated gating switches in ultra-fast central synapses.

Similar to observations in other sensory systems, developing auditory neurons display spontaneous mini-burst firings with dominant frequencies ranging from 10 to 100 Hz prior to hearing onset [2–4]. It is believed that such activity originates from the cochlea where ATP released from supporting cells evokes glutamate release from inner hair cells to activate auditory nerves and propagate activity downstream to several nuclei in the auditory brainstem [2, 17], including the MNTB [3, 29]. The prevailing view is that this spontaneous burst activity plays instructive roles in driving the organization of wiring and refinement of synapses during this highly dynamic period of development, transforming the capacity of auditory synapses to transmit at extraordinarily high rates with temporal fidelity [30]. The patterns of spontaneous firings of MNTB neurons in vivo differ strongly between prehearing and hearing mice. Before P10, spontaneous spikes appear in bursts followed by silent periods for as long as 30 s, and these silent periods diminish after P10. The TBS paradigm used in this study (i.e. 60 ms long, 50 Hz bursts with 1 s intervals for 2 min) was designed to approximate mini-burst patterns observed in pre-hearing auditory circuits with the exception for long quiescent periods. When applied in rapid succession, this paradigm can induce activation of NMDARs and Group 1 mGluRs, resulting in an extensive rise in postsynaptic Ca^2+^ to warrant the induction of synaptic plasticity. We suggested that this TBS paradigm accelerates changes in AMPAR gating switch in vitro much more rapidly than developmental remodeling process that may take days to complete in vivo.

Differing from the requirements for inducing hippocampal long-term plasticity with activation of either NMDARs or Group 1 mGluRs to recruit Ca^2+^-permeable AMPARs [31], our study shows both NMDAR and Group 1 mGluRs are essential for the gating switch at the developing calyx of Held-MNTB synapse. We have previously shown that the same TBS paradigm can lead to a rapid Ca^2+^-dependent down-regulation of extrasynaptic NMDARs via endocytosis. Results of this study reinforce the critical role of the synergistic action of NMDARs and Group 1 mGluRs in controlling the subsequent gating switch in synaptic AMPARs. The observation that the fast buffer BAPTA but not the slow buffer EGTA was able to block the induction of the gating switch in synaptic AMPARs (Fig. 5a, b) implies that Ca^2+^ signaling mediated by NMDARs and Group 1 mGluRs must be in close spatial proximity to the effector molecules to enable nanodomain coupling, reminiscent of compartmentalized Ca^2+^ signaling in dendrite spines [32]. Indeed, Group 1 mGluRs are localized to peri−/extrasynaptic domains where NMDARs are internalized following the TBS [12, 15]. Action potential driven Ca^2+^ influx, likely thorough voltage-gated Ca^2+^ channels, may also play a role, because in the presence TTX, co-application of NMDA and DHPG failed to initiate the gating switch (Fig. 4d). It is however conceivable that the only requirement of action potentials is to transiently alleviate the Mg^2+^ block of NMDARs and facilitate Ca^2+^ influx. Although synaptic AMPARs are Ca^2+^ permeable at the calyx of Held-MNTB synapse, co-application of NMDA and DHPG without AMPAR activation is sufficient to induce the gating switch (Fig. 4c), suggesting Ca^2+^ influx via AMPARs is not necessary [8, 9, 33]. Taken together, we suggest a tightly regulated surge in intracellular Ca^2+^ via a route-specific manner is required for initiating downstream Ca^2+^-dependent signaling to trigger the gating switch in synaptic GluA subtypes.

It has been well established in many central synapses that expression of long-term synaptic plasticity depends on Ca^2+^-dependent activation of CaMKII and PKC signaling [13], both of which are evidently involved in the gating switch of synaptic AMPARs at the developing calyx of Held-MNTB synapse. In hippocampal slices, brief application of Group 1 mGluR agonists activates CaMKII resulting in rapid translation of the immediate early gene product Arc/Arg3.1 which is targeted to synapses to initiate GluA1 internalization within 1 h of stimulation [27, 34, 35]. Given the necessity of dynamin in promoting NMDAR endocytosis at this synapse [12], it is possible that a similar Arc/Arg3.1 dependent endocytosis of GluA1 is likely at play, as implicated by our observation that the relative weight of two cohorts of mEPSCs (i.e. μ_slow_ = 0.8 ms vs. μ_Fast_ = 0.4 ms) decreases. PKC signaling is potentially involved in GluA4 exocytosis since it has been shown that phosphorylation of GluA4 by the PKCγ subtype is sufficient to drive the membrane insertion of these AMPARs [36]. Given the long temporal delay in the expression of the functional gating switch of synaptic AMPARs and its dependence on protein synthesis, we suggest that GluA1 removal from and GluA4 recruitment to PSDs are two regulated processes occurring in parallel. Several studies in other preparations (e.g. hippocampus) have demonstrated long-term synaptic plasticity can be accounted for by the early activity-dependent recruitment of AMPARs, particularly Ca^2+^ permeable AMPARs, immediately following the induction stimulus, and by changes in the constitutive recycling of other types of AMPARs in the late phase [13]. At the calyx of Held-MNTB synapse, we observed seemingly concurrent insertion of GluA4 and removal of GluA1 only after a prolonged post-induction period (Fig. 1e, f). Given that peri−/extrasynaptic NMDARs are internalized within the first 20–30 min following TBS [12], we postulate that this delayed expression of the gating switch of AMPARs may be a highly programmed step following the NMDAR clearance before trafficking of GluA1 and GluA4 can commence in this specialized region [37, 38].

To circumvent confounding presynaptic factors, we have analysed the decay kinetics of mEPSCs, from which we can infer the composition of synaptic AMPARs at individual postsynaptic densities of this axosomatic synapse, where the time course of mEPSCs can be reliably measured without cable filtering distortion associated with typical dendritic spine synapses. The distinctive bimodal distribution pattern of time constants for mEPSCs in the same synapses suggests the existence of at least two PSD populations within the same synapse. On the basis of previous work showing little contributions from GluA2/3 at the developing calyx of Held synapse [8, 11], we postulate that the decay time course of mEPSCs is governed by two populations of PSDs harboring homomeric channels with slow-gating GluA1 and fast-gating GluA4 AMPAR clusters (μ_slow_ = 0.8 and μ_Fast_ = 0.4 ms), respectively. If GluA1 and GluA4 can form heteromeric channels, one would expect that the distribution pattern of mEPSC τ_fast_ values be a skewed continuum instead of that with two distinct peaks. This notion can be rationalized by a complete disappearance of μ_Fast_ = 0.4 ms population in GluA4^−/−^ synapses where the μ_slow_ = 0.8 ms cohort, likely the correlate of GluA1 homomers, remained intact.

**Fig. 7:**
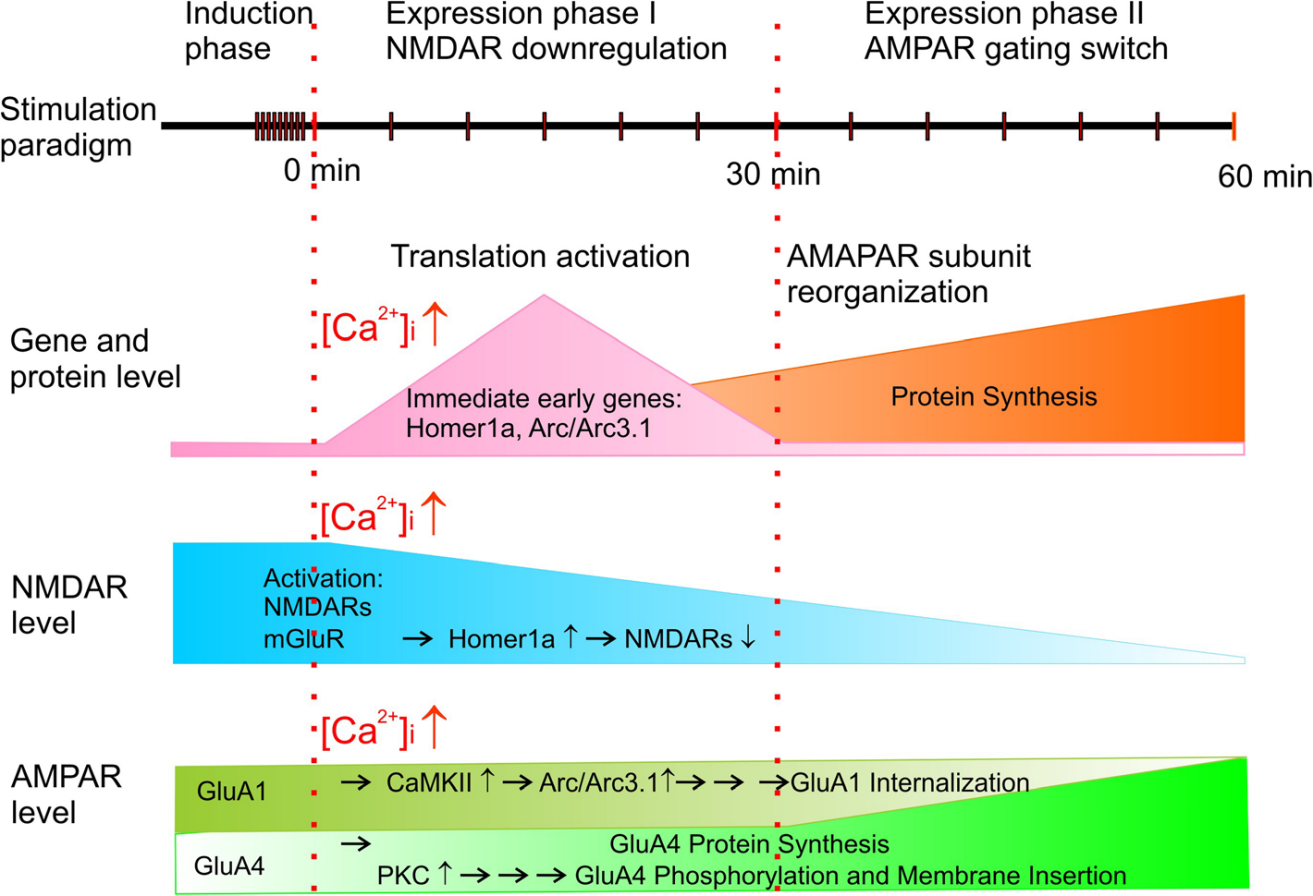
Working model for the delayed expression of activity-dependent gating switch in NMDARs and AMPARs. During *Induction Phase*, theta burst stimulation increases intracellular Ca^2+^ concentration ([Ca^2+^]_i_) via coincident activation of NMDARs and mGluR1/5 at the developing calyx of Held synapse. The rise in [Ca^2+^]_I_,causes translation activation of immediate early genes, Homer1a and Arc/Arc3.1. Homer1a can lead to a rapid internalization of extra/peri-synaptic NMDARs (*Expression Phase I*), whilst Arc/Arc3.1 proteins are phosphorylated by CaMKII to mediate internalization of GluA1 at a slower time scale (*Expression Phase II*). During *Expression Phase I*, protein synthesis builds up a new intracellular pool of GluA4 proteins that are primed by PKC-dependent phosphorylation. This new pool can be subsequently recruited to take place of synaptic slots vacated by GluA1 channels in *Expression Phase II*

Our results lead us to suggest a model (Fig. 7), in which the calyx of Held-MNTB synapse expresses both GluA1 and GluA4 AMPARs with NMDARs and Group 1 mGluRs being expressed primarily in the peri−/extrasynaptic zones prior to the onset of hearing. Spontaneous high-frequency mini-burst activity, as mimicked by TBS, stimulates the afferents to release glutamate and spill over into extrasynaptic regions, resulting in the activation of NMDARs and mGluRs. The tight spatial coupling of mGluRs and NMDARs induces a strong surge in intracellular Ca^2+^ which activates Ca^2+^-dependent translation and post-translational mechanisms to first internalize NMDARs and subsequently activate the trafficking of GluA1 and GluA4 at PSDs. An overall shift in the relative weight of faster gating GluA4 over slow gating GluA1 AMPARs ultimately accounts for the acceleration in the time course of synaptic responses at the calyx of Held-MNTB synapse. Although we did not directly test whether such a subunit switch enhances the temporal precision of spiking at different frequencies in this study, our previous work have demonstrated that accelerated decay time course of AMPAR-EPSCs alone can promote high-frequency firings of MNTB neurons independent of other confounding factors [8], and that deletion of GluA4 compromises the fidelity of neurotransmission [11]. Since GluA4 AMPARs are particularly enriched in fast-spiking neurons such as interneurons in cortical circuits, as well as principle neurons in the sensory systems of the cerebellum and brainstem, this work establishes an effective platform to model activity-dependent plasticity in Ca^2+^ permeable AMPARs, and further the understanding of the molecular signaling cascades and substrates underpinning developmental plasticity in ultra-fast central synapses.

## Data Availability

All data is available upon request.

## Abbreviations

ACSF: artificial cerebrospinal fluid
AMPAR: α-amino-3-hydroxy-5-methyl-4-isoxazolepropionic acid receptor
APV: ((2*R*)-amino-5-phosphonopentanoate)
BAPTA-AM: 1,2-Bis(2-aminophenoxy)ethane-N,N,N′,N′-tetraacetic acid tetrakis(acetoxymethyl ester)
CaMKII: Ca^2+^ /calmodulin-dependent protein kinase II
CHX: cyclohexamide
DHPG: -3,5-Dihydroxyphenylglycine
eEPSC: evoked EPSC
EGTA-AM: Tetra(acetoxymethyl Ester)
LFS: low frequency stimulation
mEPSC: miniature EPSC
mGluR: metabotropic glutamate receptor
MNTB: Medial Nucleus of the Trapezoid Body
MPEP: (2-Methyl-6-(phenylethynyl)pyridine)
NMDAR: N-methyl-D-aspartate receptor
P: Postnatal day
PKC: protein kinase C
PSD: postsynaptic density
TBS: theta burst stimulation
